# Prognostic Factors for Postoperative Chronic Pain after Knee or Hip Replacement in Patients with Knee or Hip Osteoarthritis: An Umbrella Review

**DOI:** 10.3390/jcm12206624

**Published:** 2023-10-19

**Authors:** César Fernández-de-las-Peñas, Lidiane L. Florencio, Ana I. de-la-Llave-Rincón, Ricardo Ortega-Santiago, Margarita Cigarán-Méndez, Stella Fuensalida-Novo, Gustavo Plaza-Manzano, Lars Arendt-Nielsen, Juan A. Valera-Calero, Marcos J. Navarro-Santana

**Affiliations:** 1 Department of Physical Therapy, Occupational Therapy, Physical Medicine and Rehabilitation, Universidad Rey Juan Carlos (URJC), 28922 Alcorcón, Spain; lidiane.florencio@urjc.es (L.L.F.); anaisabel.delallave@urjc.es (A.I.d.-l.-L.-R.); ricardo.ortega@urjc.es (R.O.-S.); stella.fuensalida@urjc.es (S.F.-N.); 2 Department of Health Science and Technology, Center for Neuroplasticity and Pain (CNAP), SMI, Faculty of Medicine, Aalborg University, 9220 Aalborg, Denmark; lan@hst.aau.dk; 3 Department of Psychology, Universidad Rey Juan Carlos (URJC), 28922 Alcorcón, Spain; margarita.cigaran@urjc.es; 4 Department of Radiology, Rehabilitation and Physiotherapy, Complutense University of Madrid, 28040 Madrid, Spain; gusplaza@ucm.es (G.P.-M.); juavaler@ucm.es (J.A.V.-C.); marcosjose.navarrosantana@gmail.com (M.J.N.-S.); 5 Grupo InPhysio, Instituto de Investigación Sanitaria del Hospital Clínico San Carlos (IdISSC), 28040 Madrid, Spain; 6 Department of Medical Gastroenterology, Mech-Sense, Aalborg University Hospital, 9000 Aalborg, Denmark

**Keywords:** hip, knee, pain, prognostics, osteoarthritis, replacement, arthroplasty, review

## Abstract

Knee and hip osteoarthritis are highly prevalent in the older population. Management of osteoarthritis-related pain includes conservative or surgical treatment. Although knee or hip joint replacement is associated with positive outcomes, up to 30% of patients report postoperative pain in the first two years. This study aimed to synthesize current evidence on prognostic factors for predicting postoperative pain after knee or hip replacement. An umbrella review of systematic reviews was conducted to summarize the magnitude and quality of the evidence for prognostic preoperative factors predictive of postoperative chronic pain (>6 months after surgery) in patients who had received knee or hip replacement. Searches were conducted in MEDLINE, CINAHL, PubMed, PEDro, SCOPUS, Cochrane Library, and Web of Science databases from inception up to 5 August 2022 for reviews published in the English language. A narrative synthesis, a risk of bias assessment, and an evaluation of the evidence confidence were performed. Eighteen reviews (nine on knee surgery, four on hip replacement, and seven on both hip/knee replacement) were included. From 44 potential preoperative prognostic factors, just 20 were judged as having high or moderate confidence for robust findings. Race, opioid use, preoperative function, neuropathic pain symptoms, pain catastrophizing, anxiety, other pain sites, fear of movement, social support, preoperative pain, mental health, coping strategies, central sensitization-associated symptoms, and depression had high/moderate confidence for an association with postoperative chronic pain. Some comorbidities such as heart disease, stroke, lung disease, nervous system disorders, and poor circulation had high/moderate confidence for no association with postoperative chronic pain. This review has identified multiple preoperative factors (i.e., sociodemographic, clinical, psychological, cognitive) associated with postoperative chronic pain after knee or hip replacement. These factors may be used for identifying individuals at a risk of developing postoperative chronic pain. Further research can investigate the impact of using such prognostic data on treatment decisions and patient outcomes.

## 1. Introduction

According to the Global Burden Disease 2019, osteoarthritis (OA) is highly prevalent in the knee and hip joints, and its worldwide prevalence has increased in the last decades [1]. Management of OA-related pain includes conservative (e.g., physical therapy) or surgical (e.g., joint replacement) treatment. Although knee or hip OA surgery is usually associated with positive outcomes, approximately 13% to 30% of patients receiving knee or hip replacement report postoperative pain in the first two years [2,3].

Identifying preoperative predictors of poor clinical outcomes after hip or knee replacement can alter preoperative procedures (including counselling of the patients) and postoperative rehabilitation. Several preoperative factors, clinical (e.g., pain, disability), sensory (e.g., presence of sensitization-associated or neuropathic pain symptomatology), cognitive (e.g., pain catastrophizing, kinesiophobia), or psychological (e.g., anxiety or depression), have been identified as predictors of worse outcomes after either knee or hip surgery [4,5,6,7].

Although a number of single systematic reviews have investigated existing risk factors for postoperative pain after knee or hip replacement, there is a need for a comprehensive evaluation of current available evidence. Overviews of systematic reviews, or umbrella reviews, bring together the evidence from systematic reviews on a similar topic and represent one of the highest levels of evidence [8]. Given the large body of evidence in the area of prognosis reviews for postoperative pain after knee or hip surgery, we conducted an umbrella review to summarize current evidence for prognostic factors predictive of postoperative chronic pain in patients who had received knee or hip replacement while taking into account the quality of evidence and the risk of bias in identified systematic reviews.

## 2. Methods

This review adheres to the Preferred Reporting Items for Systematic Reviews and Meta-Analyses (PRISMA) statement as much as possible [9]. The review was prospectively registered in Open Science Framework (https://osf.io/27vxy, accessed on 12 September 2023).

### 2.1. Systematic Literature Search

Electronic literature searches were conducted on MEDLINE, CINAHL, PubMed, PEDro, Cochrane Library, SCOPUS, and Web of Science from their inception to 5 August 2022. When searched databases allowed limits, searches were restricted to systematic reviews and/or meta-analyses. We also screened the reference lists of the papers that were identified in database searches. Bibliographical database search strategies were conducted with the assistance of an experienced health science librarian using the following research formula:

“Replacement” AND “Arthroplasty” AND “Arthroplasty” [Mesh]) AND (“Knee” OR “Hip” OR “Knee” [Mesh] OR “Knee Joint” [Mesh] OR “Hip” [Mesh]) AND (“Risk Factors” [Mesh] OR “prognostic factors” OR “risk factors”) AND (“postoperative pain” OR “postsurgical pain”)

We also defined our search criteria as follows:

Population:

Adults (older than 18 years) suffering from knee or hip OA who had received any type of either knee or hip replacement.

Exposure:

Any potential risk/prognostic factor for the developing of pain after surgery.

Comparator:

Not applicable.

Outcome:

Risk ratio, odds ratio, or other type of statistics, which relate the prognostic/risk factor with the development of postsurgical chronic pain (at least 6 months after surgery).

### 2.2. Eligibility Criteria

The inclusion criteria for this umbrella review included (1) systematic reviews and meta-analyses published in the English language reporting preoperative prognostic factors for postoperative chronic pain in adults receiving either hip or knee joint replacement; (2) full-text report; (3) inclusion of demographic (e.g., gender, age, weight), social (e.g., socio-economicus status, income), clinical (e.g., preoperative function, preoperative pain, quality of life, comorbidities), psychological (e.g., depression, anxiety, mental health), cognitive (e.g., kinesiophobia, catastrophism), or sensory (e.g., sensitization-associated symptomatology, neuropathic pain) variables as potential preoperative risk factors; and (4) the primary outcome should include postoperative chronic pain, defined as persistent pain at least 6 months after surgery. A systematic review was excluded if (1) it focused on intervention before surgery or any type of treatment different than surgery; (2) it did not provide information between factors and postoperative pain; and (3) it did not assess the relationship between prognostic/risk factor and pain after surgery.

### 2.3. Screening, Selection Process, and Data Extraction

Articles identified from different databases were independently reviewed by two authors. First, the duplicates were removed. Second, titles and abstracts of the articles were screened for eligibility. Third, a full-text read of potential eligible studies was conducted. Authors were required to achieve a consensus on included papers. In case of discrepancy between both reviewers, a third author participated in the process to reach the consensus and to decide whether the review should be included or not.

Data from each review/meta-analysis were extracted independently by two authors using a standardized form including design, number of studies included in the systematic review, population, prognostics factors, and whether or not there was a significant relationship between the factors and postoperative chronic pain. Both authors had to achieve a consensus on each item on the data-extraction form. If disagreements occurred, a third author made the determination.

### 2.4. Assessment of Risk of Bias (RoB)

Two researchers evaluated the risk of bias across the studies using ROBIS (Risk of Bias in Systematic reviews) [10]. The ROBIS tool includes four domains (study eligibility criteria, identification and selection of studies, data collection, and study appraisal and findings). Each domain is composed of six questions (evaluated as no information, yes, probably yes, probably no, no) and a summary to determine the risk of bias as low, unclear, or high risk. The study eligibility criteria domain analyzes if the review had a predefined objective and criteria if eligibility criteria are adequate and unambiguous for the research question and if the restrictions in eligibility criteria were appropriate. Identification and selection of the study’s domain is determined if the review search used and appropriate range of databases and electronic search sources, additional methods to identify relevant reports, adequate terms and search strategy, appropriate restrictions based on date, publication format, or language, and efforts to minimize error in selection of studies. The data collection and study appraisal domain is determined if authors made sufficient efforts to minimize error in data collection, if there were sufficient study characteristics available for both review authors and readers to be able to interpret the results, if the review used all relevant study results collected for use in the data synthesis, if risk of bias was assessed with an appropriate tool, and if authors made efforts to minimize error in risk of bias assessment.

The synthesis and findings risk of bias was determined if the synthesis includes all studies that it should, predefined analyses are reported or their absence is explained, and the synthesis is appropriate in term similarity in the research questions, study designs, and outcomes across included studies.

The GRADE approach [11] was used to establish confidence in the evidence for each identified prognostic factor. Following the methods and criteria proposed by Walton et al. [12], high confidence is provided to those prognostic factors for which consistent high-quality evidence is presented with at least one high-quality systematic review (low RoB) and no conflicting systematic reviews. Moderate confidence is provided when there are consistent findings from at least one medium-quality systematic review (moderate RoB) and the majority of findings from concurrent systematic reviews (where applicable) in the same direction of effect. Low confidence is provided to a predictor when summary findings are from low or unclear RoB from the majority of systematic reviews and with conflicting results or when only a single systematic review reported significant but moderate-level findings for that particular predictor. Very low confidence is provided when none of the above conditions are met.

## 3. Results

### 3.1. Study Selection

**Figure 1:**
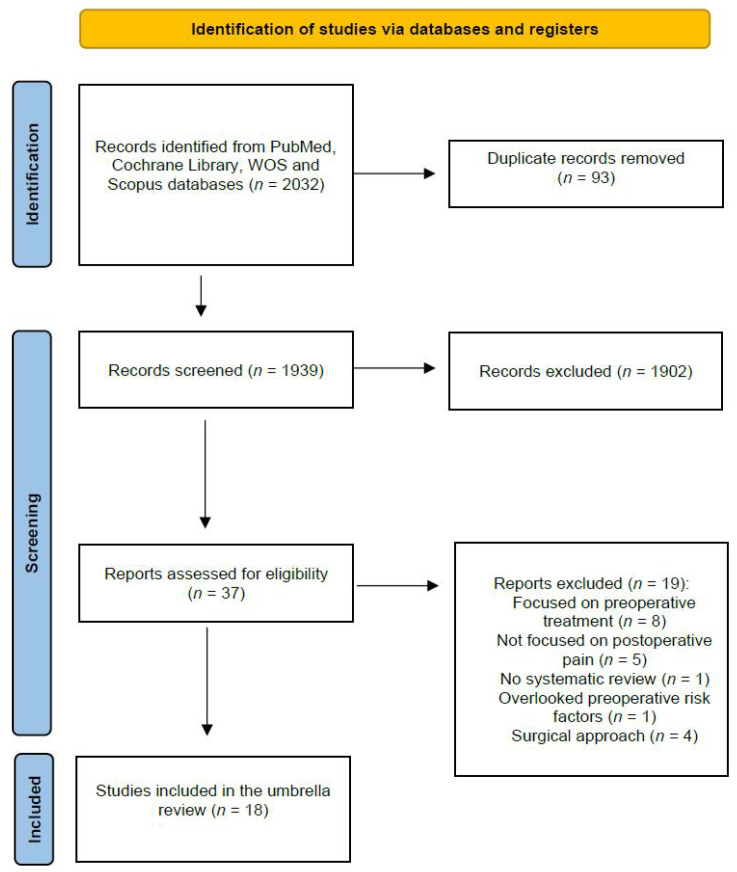
Preferred Reporting Items for Systematic Reviews and Meta-Analyses (PRISMA) 2020 flow diagram of study selection.

The electronic searches identified 2032 potential studies for review. After eliminating duplicates, 1939 studies remained. In total, 1902 (*n* = 1902) were excluded based on examination of their titles/abstracts, leaving 37 articles for full-text analysis. Another 19 were excluded because they focused on preoperative treatment or postintervention efficacy or effectiveness [13,14,15,16,17,18,19,20], did not report results about postoperative chronic pain [21,22,23,24,25], had no systematic review [26], overlooked preoperative risk factors [7], or investigated different surgical approaches [27,28,29,30]. Finally, a total of 18 reviews [31,32,33,34,35,36,37,38,39,40,41,42,43,44,45,46,47,48] were included in the umbrella (Figure 1).

### 3.2. Study Characteristics

The characteristics of the participants of the included reviews are shown in Table 1. Nine systematics reviews reported prognostic factors about knee joint replacement [30,34,35,38,40,43,44,47,48], four reviews about hip replacement [25,33,39,41], and seven included both hip/knee replacements [31,32,36,37,42,45,46,48]. There were 44 possible preoperative prognostic factors identified across studies (Table 1). Supplementary Table S1 details those prognostic factors of postsurgical chronic pain specifically investigated after knee replacement, whereas Supplementary Table S2 shows those prognostic factors specifically investigated after hip replacement. Finally, Supplementary Table S3 shows those prognostic factors investigated in reviews without differentiating between knee/hip replacement.

### 3.3. Sociodemographic Prognostic Factors

The following nine sociodemographic factors were evaluated across the studies: age, BMI, weight, sex, education level, socioeconomic status, income, employment, social support, and race. Six (6/18, 33.3%) reviews [33,37,40,41,42,43] evaluated age (two studies in knee replacement [40,43], two reviews in hip replacement [33,41], and two did not distinguish between hip/knee replacement [37,42]), seven (7/18, 38.8%) reviews [31,33,39,40,41,42,43] evaluated BMI or weight (three in knee replacement [31,40,43], four in hip replacement [31,33,39,41], and two without distinction between knee/hip [31,42]), six (6/18, 33.3%) reviews [33,40,41,42,43,45] evaluated sex (three in knee replacement [31,40,43], two in hip replacement [41,45], and one without distinction between knee/hip [42]), four (4/18, 22.2%) reviews [40,41,42,43] evaluated education level (two in knee replacement [40,43], one in hip replacement [41], and one without distinction between knee and hip [42]), two (2/18, 11.1%) reviews [40,42] evaluated socioeconomic status (one in knee [40] and one without distinction between knee/hip [42]), one (1/18, 5.5%) review evaluated monetary income and employment in knee replacement [40], four (4/18, 22.2%) reviews [38,40,42,43] evaluated social support (three in knee replacement [38,40,43] and one in knee and hip [42]), and the last one (1/18, 5.5%) evaluated patient race in a sample without distinction between knee and hip replacement [42].

### 3.4. Clinical Prognostic Factors

The following 23 preoperative clinical prognostic factors were evaluated across the studies: comorbidities (e.g., kidney disease, diabetes mellitus, heart disease, stroke), preoperative opioid use, radiographic severity, waiting list, other pain sites, preoperative function, preoperative pain, preoperative quality of life, preoperative quadriceps muscle force, preoperative flexion contracture, preoperative range of motion, neuropathic pain symptoms, central sensitization-associated symptoms, and diagnosis. Five (5/18, 27.7%) reviews [31,40,41,42,43] evaluated medical comorbidities (three in knee replacement [31,40,43], one in hip replacement [41], and one without distinction [42]), two (2/18, 11.1%) reviews [36,40] (one in knee replacement [40] and one without distinction between knee/hip [36]) evaluated medical comorbidities such as kidney disease or diabetes mellitus, one (1/18, 5.5%) review [36] evaluated other medical comorbidities such as heart disease, stroke, nervous system, lung disease, and poor circulation, one (1/18, 5.5%) review in hip/knee replacement [32] evaluated opioid use, two (2/18, 11.1%) reviews [40,41] evaluated radiographic severity (one in knee [40] and one in hip [41] replacement), one (1/18, 5.5%) review [42] in hip/knee evaluated waiting list, two (2/18, 11.1%) reviews in knee replacement [40,43] evaluated other pain sites, one (1/18, 5.5%) review in knee surgery [42] evaluated low back pain, three (3/18, 16.6%) reviews [41,42,43] (two in knee replacement [41,43] and one without distinction between knee and hip [42]) evaluated preoperative function, six (6/18, 33.3%) reviews [33,40,41,42,43,48] (three in knee replacement [41,43,48], two in hip replacement [33,41], and one without distinction [42]) evaluated preoperative pain, five (5/18, 27.8%) reviews [33,40,41,42,43] evaluated preoperative quality of life (two in knee replacement [41,43], two in hip replacement [33,41], and one without distinction between knee and hip [42]), one (1/18, 5.5%) review in both hip/knee replacement [42] evaluated waiting list, one (1/18, 5.5%) review in knee replacement [40] evaluated preoperative quadriceps muscle force, preoperative flexion contracture, and preoperative range of motion, two (2/18, 11.1%) reviews in knee replacement [34,38] evaluated neuropathic pain symptoms, three (3/18, 16.6%) reviews [35,41,48] evaluated central sensitization-associated symptoms (two in knee [35,48] and one in hip [41] replacement), and one (1/18, 5.5%) review [42] evaluated diagnosis in people without distinction between knee and hip replacement.

### 3.5. Psychological Prognostic Factors

The following 12 preoperative prognostic psychological factors were evaluated: mental health, pain catastrophizing, depression, anxiety, coping, personality, emotionally, fear of movement, self-efficacy, purpose of life, psychological distress, and expectations. Five (5/18, 27.8%) reviews [41,42,43,46,47] evaluated mental health (three in knee [43,46,47], one in hip [41], and one without distinction [42]), seven (7/18, 33.9%) reviews [38,42,43,44,46,47,48] evaluated pain catastrophizing (six in knee replacement [38,43,44,46,47,48] and one without distinction between knee/hip [42]), seven (8/18, 44.4%) reviews [36,38,40,42,43,46,47,48] evaluated depression (six in knee [38,40,43,46,47,48] and two without distinction between knee/hip [36,42]), seven (7/18, 38.9%) reviews [38,40,42,43,46,47,48] evaluated anxiety (six in knee replacement [38,40,43,46,47] and one without distinction between knee/hip replacement [42]), three (3/18, 16.6%) reviews in knee replacement [38,46,48] evaluated coping strategies, two (2/18, 11.1%) reviews [42,46] evaluated personality (one in knee [46] and one without distinction between knee/hip replacement [42]), two (2/18, 11.1%) reviews in knee replacement [46,47] evaluated emotionally, two (3/18, 16.6%) reviews in knee [38,46,48] evaluated fear of movement, two (2/18, 11.1%) reviews [42,46] evaluated self-efficacy (one in knee replacement [46] and one without distinction between knee and hip replacement [42]), one (1/18, 5.5%) review in knee replacement evaluated psychological distress [40], and one review without distinction between hip/knee [42] (1/18, 5.5%) evaluated patient expectations.

### 3.6. Risk of Bias

**Figure 2:**
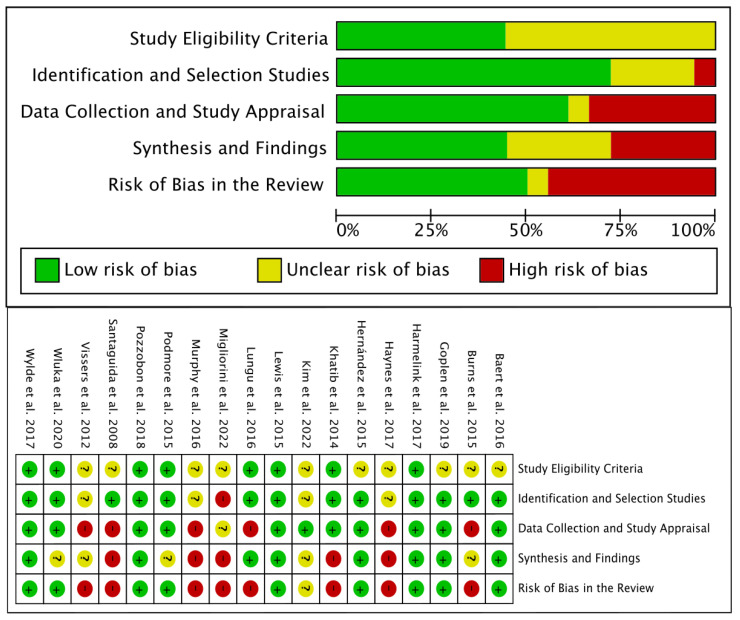
Risk of Bias (RoB) assessment using the GRADE tool [31,32,33,34,35,36,37,38,39,40,41,42,43,44,45,46,47,48].

Eight (8/18, 44.5%) reviews [33,37,39,41,44,45,46,47] were considered of high RoB, one (1/18, 5.5%) review [35] was considered of unclear/moderate RoB, and the remaining nine (9/18, 50%) reviews [31,32,34,36,38,40,42,43] of low RoB (Figure 2).

Study eligibility criteria were unclear in ten (10/18, 55.5%) reviews [32,33,35,37,39,42,44,45,46,48] and of low RoB in the remaining eight (44.4%) [31,34,36,38,40,41,43,47]. Identification and selection studies domain was of high RoB in one (1/18, 5.5%) review [33], unclear in four (4/18, 22.2%) [35,37,39,46] reviews, and of low RoB in the remaining thirteen reviews (13/18, 72.2%) [31,32,34,36,38,40,41,42,43,44,45,47,48]. The data collection and study appraisal domain was considered as high RoB in six (6/18, 33.33%) reviews [37,39,41,44,45,46], unclear in one (1/18, 5.55%) [33], and of low RoB in the remaining eleven (11/18, 61.1%) [31,32,34,35,36,38,40,42,43,47]. Synthesis and findings were of high RoB in five (5/18, 27.8%) reviews [33,37,39,45,47], unclear in five [34,35,36,44,46] (5/18, 27.8%), and of low RoB in the remaining eight reviews (8/18, 44.4%) [31,32,38,40,41,42,43].

### 3.7. Synthesis of Results

Table 2 summarizes each prognostic factor with high or moderate evidence. A total of twenty prognostic factors were identified as high or moderate confidence where the association or lack of was robust. Five prognostic factors (5/44, 9.1%) such as heart disease, stroke, lung disease, nervous system disorders, and poor circulation showed high and/or moderate confidence for no association with postoperative chronic pain. Since these prognostic factors were all just investigated in one review [36], we pooled them as comorbidities in Table 2. Accordingly, eight factors (8/44, 18.2%) including race, comorbidities, opioid use, preoperative function, sensitization-associated symptoms, neuropathic pain, pain catastrophizing, and anxiety were factors with a high confidence for a robust association, whereas seven (7/44, 15.9%) factors including other pain sites, social support, preoperative pain, mental health, depression, coping strategies, and fear of movement were factors with moderate confidence for association.

The remaining 24 factors (24/44, 54.5%) including age, BMI, sex, diagnosis, diabetes mellitus, kidney disease, radiographic severity, low back pain, contralateral hip osteoarthritis, diagnosis, level of education, socioeconomic status, income, waiting list, preoperative quality of life, preoperative quadriceps muscle force, preoperative flexion contracture, preoperative range of motion, personality, purpose in life, emotionally, self-efficacy, psychological distress, and patient expectations showed a low confidence for an association with postoperative chronic pain (Supplementary Table S4).

Comorbidities include heart disease, stroke, lung disease, nervous system disorders, and poor circulation.

## 4. Discussion

### 4.1. Findings

This umbrella review identified 18 systematic reviews summarizing the evidence for preoperative prognostic factors for postoperative chronic pain in individuals receiving knee or hip replacement. We identified 44 prognostic preoperative factors potentially associated with postoperative chronic pain, from which just 19 were judged as having high/moderate confidence for robust findings. Sixteen of these factors (i.e., race, opioid use, preoperative function, neuropathic pain symptoms, pain catastrophizing, other pain sites, anxiety, fear of movement, coping strategies, social support, preoperative pain, central sensitization-associated symptoms, mental health, depression) were associated with postoperative chronic pain, whereas five specific comorbidities (i.e., heart disease, stroke, lung disease, nervous system disorders, poor circulation) were not associated with postoperative chronic pain. The included reviews were heterogeneous in settings, prognostic factors investigated, overall quality, and follow-up periods, thus, accordingly, pooling data was not possible.

### 4.2. Sociodemographic Preoperative Factors

African-American race and lower social support were sociodemographic factors with high/moderate confidence of association with postoperative chronic pain after hip and knee replacement. The biopsychosocial model of pain would support the role of lower social support and race for the development and perpetuation of chronic pain [49,50]. It is possible that healthcare system situations, e.g., African-American patients tend to attend to medical doctors to a lower extent due to poor healthcare access, or biological, e.g., African-American patients present longer delay to presentation of the OA condition, factors explain these associations with postoperative chronic pain. From these two factors, social support is a potential but difficult modifiable factor. In such a scenario, healthcare systems should consider social and economic situations of these patients and can promote social and economic sources for these cases.

Although older age, female sex, and overweight are factors generally considered to be associated with knee and hip OA, this review showed low confidence of association of these factors with postoperative chronic pain. These results disagree with those observed by different reviews reporting that female sex, younger age, and higher BMI were associated with overall postoperative chronic pain [51]. Younger age and overweight were factors associated with higher postoperative chronic pain in breast cancer and thoracic surgery [52]. These discrepancies may be related to the fact that knee and hip replacements are recommended to be used in people older than 60 years, whereas other surgical procedures are applied in a large range of age. Accordingly, it is possible that the effect of age could be limited in surgical procedures where the age range of the patients is narrower.

### 4.3. Clinical and Sensory Preoperative Factors

Preoperative pain and function and other pain sites were clinical factors associated with higher postoperative chronic pain after hip/knee replacement. These results agree with those reported by Andreoletti et al. [53] and Yang et al. [51], who also observed that preoperative pain intensity and the presence of preoperative pain elsewhere (other pain sites) were overall associated with postoperative chronic pain. A higher level of preoperative pain was also associated with postoperative chronic pain in breast and thoracic surgery [52]. In agreement with current data, our umbrella review also found that preoperative pain and function status are relevant for the development of postoperative chronic pain after knee or hip replacement. In such a scenario, preoperative interventions aiming to improve pain and function in these patients could help with postoperative chronic pain, although evidence is limited [54].

The presence of widespread pressure pain hyperalgesia (a sign of sensitization) is associated with the development of musculoskeletal pain [55], with a negative outcome after conservative treatment in musculoskeletal pain conditions, including knee and hip OA [56], and with postoperative chronic pain [4]. These findings agree with the results from this umbrella review since the presence of central sensitization-associated symptoms was associated with postoperative chronic pain. The role of preoperative sensitization-associated symptoms with postoperative chronic pain is supported by the effects of ketamine as a preventive medication for postoperative chronic pain [57]. However, it has been recently observed that the effect of preoperative administration of ketamine in postoperative opioid consumption is small [58]. In addition, the association of other pain sites (which is a sign of sensitization) with postoperative chronic pain also supports that those patients with knee/hip OA presenting features consistent with pain sensitization [59] are at a higher risk of developing postoperative chronic pain. Therefore, identification of the presence of sensitization in patients with hip or knee OA [60] who will undergo joint surgery replacement and its proper management could lead to better postoperative outcomes. In fact, a randomized clinical trial found some benefit of applying pain neuroscience education preoperatively in a sample of patients with knee OA [61].

We also found that preoperative opioid use was another factor associated with worse postoperative chronic pain. Yang et al. [51] observed that preoperative analgesia use (not necessarily opioid) was associated with postoperative chronic pain. Yerneni et al. found that preoperative opioid use was associated with worse postoperative outcomes in people who received spine surgery [62]. Similarly, Hannon et al. found that preoperative opioid use leads to more complications after knee replacement, higher risk of postoperative chronic opioid use, and worse patient-reported outcomes [63]. Accordingly, current data support that preoperative opioid use is associated with worse postoperative outcomes. In such a scenario, higher preoperative pain intensity, the presence of neuropathic pain, or the presence of other pain sites (as expression of sensitization) could explain a higher use of opioids preoperatively; therefore, a public healthcare decision for decreasing preoperative analgesia prescription and use, particularly opioids, could be considered. For instance, it is possible that preoperative analgesia is related to long-term waiting lists for receiving surgery; accordingly, early surgery could reduce its use. Additionally, the use of non-pharmacological analgesic therapeutic strategies could be also implemented.

### 4.4. Psychological and Cognitive Preoperative Factors

A recent meta-analysis concluded that anxiety/depressive levels, mental health, catastrophizing, and, to a lesser extent, kinesiophobia showed significant association with postoperative chronic pain [64]. In fact, the fact that preoperative anxiety/depressive levels are associated with postoperative chronic pain has been also confirmed by previous meta-analyses [51,53]. In agreement, the current umbrella review identified that anxiety, depression, mental health, pain catastrophizing, coping strategies, and kinesiophobia were factors showing high or moderate confidence of association with postoperative chronic pain in patients receiving knee/hip replacement. In fact, Fonseca-Rodrigues et al. identified a significant correlation between pain intensity and depressive/anxiety levels in patients with OA [65]. This association is further confirmed by the fact that preoperative chronic pain and function and preoperative levels of anxiety/depression were factors associated with postoperative pain in individuals receiving hip or knee surgery.

In addition to mood disorders, we also found that cognitive factors related to pain experience, e.g., kinesiophobia, pain catastrophizing, or coping strategies, were associated with postoperative chronic pain after knee or hip joint replacement. In agreement with our results, cognitive factors such as catastrophic thinking and self-efficacy have been also found to be indicators of poor postoperative outcomes in people with carpal tunnel syndrome who receive surgery [66] and also in women after breast cancer surgery [67]. Thus, treatments should target and optimize these modifiable factors since a potential increased focus on positive psychological protective factors may provide better postoperative outcomes [68].

### 4.5. Limitations

To the best of our knowledge, no other umbrella systematic review is available on the topic of postoperative chronic pain after knee or hip replacement. First, it is important to note that the current umbrella review included a large quantity of reviews and a critical analysis of the quality of the reviews; however, due to the heterogeneity of the included systematic reviews in the follow-up periods, the risk models involved, and the outcomes reported, a multiple meta-analysis was not possible to conduct. In fact, multiple meta-analysis is usually conducted for treatment approaches and not for prognostics factors such as those included in our umbrella review [69]. Second, the methodological quality of the reviews, as assessed with the ROBIS tool, was also heterogeneous, including high-quality and low-quality reviews. Third, it should be pointed out that the reviews evaluated in this umbrella review are not primary studies and therefore could be affected by some bias. In fact, almost 50% of the reviews showed high RoB. Finally, it is possible that the same evidence has been considered multiple times throughout different reviews, an inherent limitation of umbrella reviews. Finally, we did not search in some other databases such as EMBASED, and we just restricted the current analysis to studies published in the English language.

## 5. Conclusions

This umbrella review identified multiple preoperative factors (i.e., sociodemographic, clinical/sensory, or psychological/cognitive) associated with postoperative chronic pain after knee or hip replacement. Since most of the identified associated factors are modifiable, they can be used for identifying individuals at a risk of developing postoperative chronic pain and could be preoperatively managed to investigate the impact of using such prognostic data on treatment decisions and patient outcomes. For instance, managing preoperative pain-related factors (e.g., sensitization-associated and neuropathic pain symptoms) and function could reduce the development of postoperative chronic pain. Similarly, since some factors are sociocultural and economic, health care systems should be also involved in the management of these patients.

## Figures and Tables

**Table 1:** Characteristics of included systematic reviews or meta-analyses.

Author	Type of Study	Characteristics of the Sample	Studies Included	Prognostic Factors Analyzed
Santaguida et al. [45]	Systematic review	JKR or JHR	64 studies (6 prospective studies, 58 retrospective studies)	Age; sex; BMI or weight
Vissers et al. [46]	Systematic review	TKA and THA	35 prospective studies	Mental health; pain catastrophizing; depression; anxiety; coping; personality; perceived stress; emotionality; fear of movement; sense of coherence; fatigue
Khatib et al. [47]	Systematic review	KJR	17 prospective cohort studies and 2 cross-sectional surveys	Mental health; anxiety; depression; pain catastrophizing; self-efficacy; quality of life
Burns et al. [44]	Systematic review	TKA	6 studies (2 prospective cohort studies, 3 case-control studies, and 1 cohort and case-control study)	Pain catastrophizing
Hernández et al. [42]	Systematic review	Total hip and knee arthroplasty	37 studies (4 systematic reviews, 1 randomized clinical trial, and 32 observational studies)	Age; sex; level of education; socioeconomic status; social support; preoperative educational interventions; race; body mass index; comorbidities; preoperative pain; preoperative functional capacity; self-efficacy; patient expectations; pain catastrophizing; depression; anxiety; personality; mental health; other psychological factors
Lewis et al. [43]	Systematic review and meta-analysis	TKA	32 studies (28 studies included in meta-analyses, 23 prospective studies)	Age; weight; social support; anxiety; pain catastrophizing; comorbidities; depression; education; gender; mental health; other pain sites; preoperative pain
Lungu et al. [41]	Systematic review	THA	22 studies included	Age; sex; BMI; lower educational; surgery expectations; preoperative pain; preoperative functional capacity; mental health; physical health; physical status; comorbidities; knee extensor strength; radiographic OA severity; waiting time for surgery; widespread pain sensitivity
Baert et al. [48]	Systematic review	TKA	16 studies included (cohort studies)	Depressive symptoms; anxiety, pain catastrophizing; fear of movement; coping strategy; preoperative pain; widespread pain sensitivity
Harmenlink et al. [40]	Systematic review	TKA from OA with a follow-up > 1 yr	18 studies (13 prospective studies, 5 retrospective studies)	Age; sex; BMI; anxiety; depression; social support; education; income; employment; preoperative pain; physical function; preoperative quadriceps muscle force; preoperative flexion contracture; preoperative ROM; quality of life; comorbidities; walking distance; painful other joints; radiographic severity; overall health status
Wylde et al. [38]	Systematic review	TKR and follow-up less than 6 months	14 studies (11 cohort, 2 RCT, 2 retrospective, and 1 case-control)	Pain catastrophizing; depression; anxiety; social support; coping strategies; fear of movement
Haynes et al. [39]	Systematic review	THA	17 cohort studies	BMI
Goplen et al. [32]	Systematic review and metanalysis	Total joint arthroplasty	6 retrospective studies	Preoperative opioid use
Wluka et al. [34]	Systematic review and metanalysis	TKR	5 manuscripts with 6 cohorts (4 studies, 1 pre-pint)	Neuropathic-like pain symptoms
Podmore et al. [36]	Systematic review and metanalysis	TKR and THR	70 observational studies	Depression, comorbidities
Murphy et al. [37]	Systematic review	TKA and THA	32 studies included	Age
Pozzobon et al. [31]	Systematic review and metanalysis	TKA and THA	62 studies included in the qualitative analysis. 31 in the meta-analyses	BMI
Migliorini et al. [33]	Systematic review and metanalysis	Minimal invasive THA	76 studies (33 randomized clinical trials, 29 prospective studies, and 17 retrospective studies)	Sex; age; BMI; preoperative pain, preoperative function
Kim et al. [35]	Systematic review and meta-analysis	TKA	8 studies in the systematic review (5 prospective studies, 1 randomized clinical trial, and 2 retrospective studies). 5 studies in the meta-analyses	Central sensitization (CSI)

TKA: total knee arthroplasty; THA: total hip arthroplasty; JKR: joint knee replacement; JHR: joint hip replacement; ROM: range of motion.

**Table 2:** Prognostic factors of postsurgical pain with high/moderate confidence in conclusions.

Prognostic Factor	Reviews Investigating a Particular Prognostic Factor	Overall Risk of Bias (ROBIS)			Summary of Findings from Reviews			Confidence in Conclusions Based on All Review Findings
		Low	Unclear	High	Consistent (High, Moderate, Low, or Very Low): Association	Consistent (Moderate/Strong Evidence): NO Association	Conflicting/Inconsistent or Weak/Limited Evidence	High/Moderate/Low/Very Low
Race	One review (Hernández et al. [42])	1/1 (Hernández et al. [42])			1 (Hernández et al. [42])			High confidence: worse postoperative pain (1 low RoB)
Comorbidities	One review (Podmore et al. [36])	1/1 (Podmore et al. [36])				1/1 (Podmore et al. [36])		High confidence: no association (1 low RoB)
Other pain sites	Two reviews (Harmelink et al. [40], Lewis et al. [43])	2/2 (Harmelink et al. [40], Lewis et al. [43])			1 (Lewis et al. [43])		1 (Harmelink et al. [40])	Moderate confidence: worse postoperative pain—1 review (1 low RoB) and 1 very low evidence for association (1 low RoB)
Opioid use	One review (Goplen et al. [32])	1/1 (Goplen et al. [32])			1 (Goplen et al. [32])			High confidence: worse postoperative pain (1 low RoB)
Social support	Four reviews (Harmelink et al. [40], Hernández et al. [42], Lewis et al. [43], Wylde et al. [38])	4/4 (Harmelink et al. [40], Hernández et al. [42], Lewis et al. [43], Wylde et al. [38])			2/4 (Lewis et al. [43], Wylde et al. [38])		2/4 (Harmelink et al. [40], Hernández et al. [42])	Moderate confidence: worse postoperative pain—2 reviews (2 low RoB), 1 reported conflicting evidence (low RoB), and 1 reported very low evidence of association (low RoB)
Preoperative function	Three reviews (Hernández et al. [42], Lewis et al. [43], Lungu et al. [41])	2/3 (Hernández et al. [42], Lewis et al. [43])		1/3 (Lungu et al. [41])	3 (Hernández et al. [42], Lewis et al. [43], Lungu et al. [41])			High confidence: worse postoperative pain—3 reviews (2 low RoB and 1 high RoB)
Preoperative pain	Six reviews (Baert et al. [48], Harmelink et al. [40], Hernández et al. [42], Lewis et al. [43], Lungu et al. [41], Migliorini et al. [33])	4/6 (Harmelink et al. [40], Hernández et al. [42], Lewis et al. [43])		2/6 (Lungu et al. [41], Migliorini et al. [33])	4 (Hernández et al. [42], Lewis et al. [43], Lungu et al. [41], Migliorini et al. [33])		2 (Baert et al. [48], Harmelink et al. [40])	Moderate confidence: worse postoperative pain—4 reviews (2 low RoB) and 2 reported very low evidence for association (low RoB)
Central sensitization	Three reviews (Baert et al. [48], Kim et al. [35], Lungu et al. [41])	1/3 (Baert et al. [48])	1/3 (Kim et al. [35])	1/3 (Lungu et al. [41])	3 (Kim et al. [35], Baert et al. [48], Lungu et al. [41])			High confidence: worse postoperative pain—3 reviews (1 low RoB, 1 unclear RoB, and 1 high RoB)
Neuropathic pain	Two reviews (Wluka et al. [34], Wylde et al. [38])	2/2 (Wluka et al. [34], Wylde et al. [38])			2 (Wluka et al. [34], Wylde et al. [38])			High confidence: worse postoperative pain—2 reviews (2 low RoB)
Mental health	Five reviews (Hernández et al. [42], Khatib et al. [47], Lewis et al. [43], Lungu et al. [41], Vissers et al. [46])	2/5 (Hernández et al. [42]. Lewis et al. [43])		3/5 (Khatib et al. [47], Lungu et al. [41], Vissers et al. [46])	4 (Hernández et al. [42], Lewis et al. [43], Lungu et al. [41], Vissers et al. [46])	1 (Khatib et al. [47])		Moderate confidence: 4 reviews reporting association (2 low RoB) and 1 reporting for no association (high RoB)
Pain catastrophizing	Seven reviews (Baert et al. [48], Burns et al. [44], Hernández et al. [42], Khatib et al. [47], Lewis et al. [43], Vissers et al. [46], Wylde et al. [38])	4/7 (Baert et al. [48], Hernández et al. [42], Lewis et al. [43], Wylde et al. [38])		3/7 (Burns et al. [44], Khatib et al. [47], Vissers et al. [46])	7 (Baert et al. [48], Burns et al. [44], Hernández et al. [42], Khatib et al. [47], Lewis et al. [43], Vissers et al. [46], Wylde et al. [38])			High confidence: worse postoperative pain—7 reviews reporting association (4 low RoB and 3 high RoB)
Depression	Eight reviews (Baert et al. [48], Harmelink et al. [40], Hernández et al. [42], Khatib et al. [47], Lewis et al. [43], Podmore et al. [36], Vissers et al. [46], Wylde et al. [38])	6/8 (Baert et al. [48], Harmelink et al. [40], Hernández et al. [42], Lewis et al. [43], Podmore et al. [36], Wylde et al. [38])		2/8 (Khatib et al. [47], Vissers et al. [46])	5 (Hernández et al. [42], Khatib et al. [47], Lewis et al. [43], Vissers et al. [46], Wylde et al. [38])	1 (Podmore et al. [36])	2 (Baert et al. [48], Harmelink et al. [40])	Moderate confidence: 5 reviews reporting association (3 low RoB), 1 reporting no association (1 low RoB) and 2 reported very low evidence for association (2 low RoB)
Anxiety	Seven reviews (Baert et al. [48], Harmelink et al. [40], Hernández et al. [42], Khatib et al. [47], Lewis et al. [43], Vissers et al. [46], Wylde et al. [38])	5/7 (Baert et al. [48], Harmelink et al. [40], Hernández et al. [42], Lewis et al. [43], Wylde et al. [38])		2/7 (Khatib et al. [47], Vissers et al. [46])	6 (Harmelink et al. [40], Hernández et al. [42], Khatib et al. [47], Lewis et al. [43], Vissers et al. [46], Wylde et al. [38])		1 (Baert et al. [48])	High confidence: 7 reviews reporting association (4 low RoB and 2 high RoB), and 1 reported conflicting evidence (1 low RoB)
Fear of movement	Three reviews (Baert et al. [48], Vissers et al. [46], Wylde et al. [38])	2/3 (Baert et al. [48], Wylde et al. [38])		1/3 (Vissers et al. [46])	2 (Vissers et al. [46], Wylde et al. [38])	1 (Baert et al. [48])		Moderate confidence: 2 review reporting association (1 low RoB and 1 high RoB), and 1 reported no association (1 low RoB)
Coping strategy	Three reviews (Baert et al. [48], Vissers et al. [46], Wylde et al. [38])	2/3 (Baert et al. [48], Wylde et al. [38])	0	1/3 (Visser et al. [46])	2 (Baert et al. [48], Vissers et al. [46])	1 (Wylde et al. [38])		Moderate confidence: 2 reported association (1 high RoB and 1 low RoB) and 1 review reporting no association (1 low RoB).

BMI: body mass index; RoB: risk of bias.

## Data Availability

All data derived from the study are reported in this article.

## Supplementary Materials

The following supporting information can be downloaded at: https://www.mdpi.com/article/10.3390/jcm12206624/s1, Table S1: Synthesis of prognostic factors for postoperative pain after knee replacement; Table S2: Synthesis of prognostic factors for postoperative pain after hip replacement; Table S3: Synthesis of prognostic factors for postoperative pain after knee/hip replacement; Table S4: Prognostic factors of postsurgical pain with low confidence in conclusions.
