# Supplementary material for: Prognostic Factors for Postoperative Chronic Pain after Knee or Hip Replacement in Patients with Knee or Hip Osteoarthritis: An Umbrella Review
Source: J Clin Med. 2023 Oct 19;12(20):6624. doi: 10.3390/jcm12206624 (PMC10607727; doi:10.3390/jcm12206624)
Supplement: Supplementary file 1 [file jcm-12-06624-s001.zip › Suppl Table S1.pdf]

Supplementary Table S1: Synthesis of Prognostic Factors for Postoperative Pain after Knee Replacement

Variable/ Number Studies	Santaguida et al. [45]	Vissers et al. [46]	Khatib et al. [47]	Burns et al. [44]	Lewis et al. [43]	Baert et al. [48]	Harmenlink et al. [40]	Wylde et al. [38]	Pozzobon et al. [36]	Wluka et al. [34]	Kim et al. [35]
**Age** (0/2, 0.00%)	-	-	-	-	**NS** Fisher's Z -0.005 (-0.018; 0.00), p=0.396, I^2=30%		**NS** (n=3/10) (+)	-	-	-	-
**BMI or weight** (1/3. 33.3%)	-	-	-	-	**NS** Fisher's Z -0.028 (-0.009; 0.065), n=10 p=0.791, I^2=45%		**NS** (n=3/6) (+)	-	Short term 0.55 [-0.9; -0.2] p=0.002 I^2=35.48% **S** Long Term: -0.36 [-0.48; -0.25] <0.001 I^2=0.00% **S**	-	-
**Comorbidity** (0/2. 0%)	-	-	-	-	**S** Fisher's Z 0.047 (0.016; 0.441), n=11, p=0.003, I2=0		**NS** (n=5/10) (+)	-	-	-	-
**Gender** (1/3. 33.3%)	**S** W<M (n=1)	-	-	-	**NS** Fisher's Z -0.031 (-0.033; 0.582), n=16, p=0.075, I^2=29%		**NS** (n=2/10) (+)	-	-	-	-
**Education Level** (0/2, 0%)	-	-	-	-	**NS** Fisher's Z -0.029 (-0.071; 0.014), n=7 p=0.186, I^2=12%		**NS** (n=0/1) (+)	-	-	-	-
**Socioeconomic status** (0/1. 0%)	-	-	-	-	-		**NS** (n=0/2) (+)	-	-	-	-
**Income** (1/1, 100%)	-	-	-	-	-		**S** (n=1/1) (+)	-	-	-	-
**Employment before surgery** (0/1, 0%)	-	-	-	-	-		**NS** (n=0/2) (+)	-	-	-	-
**Social support** (3/3, 100%)	-	-	-	-	**NS** Fisher's Z -0.004 (-0.049; 0.041), n=4 p=0.864. I^2=0%		↓ (n=4/4) (+)	↓ (n=1/1)	-	-	-
**Radiographic severity** (1/1, 100%)			-	-	-		↓ (n=2/2) (+)	-	-	-	-
**Other pain sites** (2/2, 100%)	-	-	-	-	**NS** Fisher's Z 0.0275 (-0.033; 0582), n=4 p=0.08, I^2=88%		↑ (n=2/2) (+)	-	-	-	-
**Diabetes Mellitus** (1/1, 100%)	-	-	-	-	-		↑ (n=1/1) (+)	-	-	-	-
**Kidney disease** (1/1, 100%)	-	-	-	-	-		↓ (n=1/1) (+)	-	-	-	-
**Quality of life** (2/2, 100%)	-	-	**S** (n=3)	-	-		↑ (n=2/2) (+)	-	-	-	-
**Preoperative function** (1/1, 100%)	-	-	-	-	**NS** Fisher's Z -0.005 (-0.044; 0.033), n=5, p=0.785, I^2=0	↑ (n=1) (+)	-	-	-	-	-

**Preoperative pain** (2/2, 100%)	-	-	-	-	**S** Fisher's Z 0.159 (0.077; 0.240), n=16, p<0.001, I^2=82%	↑ (n=1/1) (+)	↑ (n=8/10) (+)	-	-	-	-
**Quadriceps muscle force** (0/1, 0%)	-	-	-	-	-		**NS** (n=0/1) (+)	-	-	-	-
**Preoperative flexion contracture** (1/1, 100%)	-	-	-	-	-		↓ (n=1/1) (+)	-	-	-	-
**Preoperative ROM** (0/1, 0%)	-	-	-	-	-		**NS** (n=0/1) (+)	-	-	-	-
**Neuropathic pain** (2/2, 100%)	-	-	-	-	-		-	**S** (n=1/1)	-	**S** RR 2.05 (1.51; 2.79) n=3	-
**Central sensitization** (2/2, 100%)	-	-	-	-	-	↑ (n=2/2) (++)	-	-	-	-	SMD: 0.65 [0.40-0.90] p<0.001 n=5 **S**
**Mental health** (2/3, 66.6%)	-	↑ **<1yr** (n=1) ↑ **>1yr** (n=2)	**NS** (n=2/8)	-	**S** Fisher's Z -0.108 (-0.148; -0.068), n=4 p<0.001, I^2=0		-	-	-	-	-
**Pain Catastrophizing** (5/5, 100%)	-	↑ **<1yr** (n=2) **NS >1yr** (n=1)	**S** (n=2)	↑ (n=5/6)	**S** Fisher's Z 0.302 (0.162; 0.441), n=2 p<0.001, I^2=0%	↑ (n=7) (+++)	-	**S** (Night pain. n=1/1) **NS** (Global pain (n=0/1)	-	-	-
**Depression** (4/5, 80%)	-	**? <1yr** (n=2) ↓**>1yr** (n=2)	**S** (n=4/4)	-	**S** Fisher's Z 0.064 (-0.005; 0.132), n=6 p=0.069, I^2=41%	**?** (n=3/10) (+)	**NS** (n=1/3) (+)	**S** (Global pain. n=1/1) **NS** (Night pain (n=0/1)	-	-	-
**Anxiety** (5/6, 100%)	-	**NS<1yr** (n=1) ↓ **>1yr** (n=1)	**S** (n=3/4)	-	**S** Fisher's Z= 0.089 (-0.090; 0.267)., n=3 p=0.330, I2=62	**?** (n=3/6) (+)	↑ (n=1/1) (+++)	**S** (n=1/1)	-	-	-
**Coping** (2/3, 66.66%)	-	↑ (<1yr, >1yr) (n=1)	-	-	-	**S** (n=2/2) (+++)	-	**NS** (n=1/1)	-	-	-
**Personality** (0/1, 0%)	-	**?** (n=2)	-	-	-		-	-	-	-	-
**Purpose in life** (1/2, 50%)	-	**NS (<1yr)** (n=1)	**S** (n=1)	-	-		-	-	-	-	-
**Emotionally** (0/1, 0%)	-	**NS (<1yr)** (n=1)	-	-	-		-	-	-	-	-
**Fear of movement** (2/3. 66.66%)	-	↓ **(<1yr)** (n=1)	-	-	**NS** (n=0/4) (+++)		-	**S** (n=1/1)	-	-	-
**Self-Efficacy** (1/1, 100%)	-	-	**S** (n=1)	-	-		-	-	-	-	-
**Psychological distress** (0/1, 0%)	-	-	-	-	-		**NS** (n=0/1) (+)	-	-	-	-

↓ Significant negative (indirect association) influence; ↑ Significant positive (direct association) influence; NS No significant influence or null/insignificance effect; S Significant influence (not specify); W Women; M Men; ? Unclear. conflicting or undetermined results; NA Not applicable; yr years; N Number of studies
