# Supplementary material for: Prognostic Factors for Postoperative Chronic Pain after Knee or Hip Replacement in Patients with Knee or Hip Osteoarthritis: An Umbrella Review
Source: J Clin Med. 2023 Oct 19;12(20):6624. doi: 10.3390/jcm12206624 (PMC10607727; doi:10.3390/jcm12206624)
Supplement: Supplementary file 1 [file jcm-12-06624-s001.zip › Suppl Table S2.pdf]

Supplementary Table S2: Synthesis of Prognostic Factors for Postoperative Pain after Hip Replacement

Variable	Santaguida et al. [45]	Vissers et al. [46]	Lungu et al. [41]	Haynes et al. [39]	Pozzobon et al. [31]	Migliorini et al. [33]
**Age** (1/2, 50%)	-	-	NS (n=4/11)			S r=0.42 p=0.02
**BMI or weight** (1/3, 33.33%)	-	-	↑ (n=6/10)	NS (N=3/11)	**Short term (<6 months)**<br>SMD: -0.34 [-0.67; -0.02] p=0.039 n=0/2 I^2=0.00%<br>**Long-term (>6 months)**<br>SMD: -0.32 [-0.84; 0.20] p=0.222 n=1/2 I^2=94.49%	NS r=0.17 p=0.4
**Comorbidity** (1/1, 100%)	-	-	↑ (n=7/8)	-	-	-
**Gender** (1/3, 33.33%)	W < M (n=1)	-	NS (n=2/9)	-	-	NS r=-0.26 p=0.2
**Level of education** (1/1, 100%)	-	-	↑ (n=3/3)	-	-	-
**Contralateral hip OA** (1/1, 100%)			S (n=1)	-	-	-
**Radiographic severity** (1/1, 100%)	-	-	↑ (n=3/3)	-	-	-
**Quality of life** (1/1, 100%)	-	-	↓ (n=4/4)	-	-	-
**Preoperative function** (2/2, 100%)	-	-	↓ (n=9/12)	-	-	S r=0.88 p=0.02
**Preoperative pain** (2/2, 100%)	-	-	↑ (n=9/12)	-	-	S r=0.98 p<0.001
**Mental Health** (2/2, 100%)	-	↑ (< and > 1 yr) (n=1)	↓ (n=2)	-	-	-
**Widespread sensitivity** (1/1, 100%)	-	-	S (n=1/1)	-	-	-

↓ Significant negative (indirect association) influence; ↑ Significant positive (direct association) influence; NS No significant influence or null/insignificance effect; S Significant influence (not specify); W: Women; M: Men; NA: Not applicable; yr: years; n: Number of studies
