# Supplementary material for: Prognostic Factors for Postoperative Chronic Pain after Knee or Hip Replacement in Patients with Knee or Hip Osteoarthritis: An Umbrella Review
Source: J Clin Med. 2023 Oct 19;12(20):6624. doi: 10.3390/jcm12206624 (PMC10607727; doi:10.3390/jcm12206624)
Supplement: Supplementary file 1 [file jcm-12-06624-s001.zip › Suppl Table S3.pdf]

Supplementary Table S3: Synthesis of Prognostic Factors for Postoperative Pain after Knee/Hip Replacement

Variable	Hernández et al. [42]	Murphy et al. [37]	Pozzobon et al. [31]	Podmore et al. [36]	Goplen et al. [32]
**Age** (1/2, 50%)	↓↓ (n=4); ↑ (n=2); NS (n=8)	**NS**	-	-	-
**BMI or weigth** (1/2, 50%)	**?** (n=4/11)	-	**Short term (<6 months)**<br>SMD: -0.44 [-0.68; -0.20]<br>P<0.001 n=1/4, I^2=0.00%<br><br>THA: -0.34 [-0.67; -0.02]<br>p=0.039 n=0/2 I^2=0.00%<br><br>**Long-term** (6 months o >)<br>SMD: -0.36 [-0.47; -0.25]<br>p<0.001 n=2/7 I^2=72.36%<br><br>THA: [-0.32 -0.84; 0.20]<br>p=0.222 n=1/2 I^2=94.49%	-	-
**Comorbidity** (0/1, 0%)	**?** (n=2/10)	-	-	-	-
**Gender** (1/1, 100%)	**W>M** (n=5/10)	-	-	-	-
**Level of education** (0/1, 0%)	**?** (n=1/6)	-	-	-	-
**Socioeconomic status** (1/1, 100%)	↓ (n=2/2)	-	-	-	-
**Social support** (0/1, 0%)	**?** (n=1/2)	-	-	-	-
**Preoperative educational intervention** (0/1, 0%)	**NS** (n=1)	-	-	-	-
**Opioid use** (1/1, 100%)	-	-	-	-	TKA: SMD -0.53<br>[-0.75; -0.32] n=6/6 **S**<br>THA: SMD=-0.26<br>[-0.56. 0.05], n=3/6 **S**
**Race** (1/1, 100%)	**S** African-American worse pain (n=2)	-	-	-	-
**Diagnosis** (0/1, 0%)	? (n=2)	-	-	-	-
**Low back pain** (1/1, 100%)	S (n=2)	-	-	-	-
**Kidney disease** (0/1, 0%)	-	-	-	OR 1.17 [0.81, 1.70] (n=2) **NS**	-
**Heart disease** (0/1, 0%)	-	-	-	OR 1.16 [0.88, 1.52] (n=2) **NS**	-
**Stroke** (0/1, 0%)	-	-	-	OR 1.41 [0.97, 2.04] (n=2) **NS**	-

**Nervous system** (0/1, 0%)		-	-	OR 1.01 [0.66, 1.54] (n=6) **NS**	-
**Lung disease** (0/1, 0%)	-	-	-	OR 1.17 [0.93, 1.46 (n=2) **NS**	-
**Poor circulation** (0/1, 0%)	-	-	-	O: 1.26 [0.98, 1.61] (n=2) **NS**	-
**Waiting list** (0/1, 0%)	**NS** (n=1)	-	-	-	-
**Preoperative function** (1/1, 100%)	↓ (n=2)	-	-	-	-
**Preoperative pain** (1/1, 100%)	↑ (n=5) ↓ (n=1) NS (n=1)	-	-	-	-
**Mental Health** (1/1, 100%)	↑ (n=3)	-	-	-	-
**Pain Catastrophizing** (1/1, 100%)	↑(n=2)	-	-	-	-
**Depression** (1/2, 50%)	S (n=5)	-	-	OR 1.22 [0.79, 1.87] (n=3) **NS**	-
**Anxiety** (1/1, 100%)	S (n=5)	-	-	-	-
**Personality** (0/1, 0%)	? (n=2)	-	-	-	-
**Self-Efficacy** (0/1, 0%)	? (n=2)	-	-	-	-
**Patient expectations** (0/1, 0%)	? (n=3)	-	-	-	-

↓ Significant negative (indirect association) influence; ↑ Significant positive (direct association) influence; NS No significant influence or null/insignificance effect; S Significant influence (not specify); W Women; M Men; NA Not applicable; yr years; N Number of studies; TKA: Total Knee Arthroplasty; THA; Total Hip Arthroplasty
