# Supplementary material for: Prognostic Factors for Postoperative Chronic Pain after Knee or Hip Replacement in Patients with Knee or Hip Osteoarthritis: An Umbrella Review
Source: J Clin Med. 2023 Oct 19;12(20):6624. doi: 10.3390/jcm12206624 (PMC10607727; doi:10.3390/jcm12206624)
Supplement: Supplementary file 1 [file jcm-12-06624-s001.zip › Suppl Table S4.pdf]

Supplementary Table S4: Prognostic Factors of Postsurgical Pain with Low Confidence in Conclusions

Prognostic factor	Reviews investigating the prognostic factor	Overall risk of bias (ROBIS)			Summary of findings from reviews			Confidence in conclusions based on all review findings
		Low	Unclear	High	Consistent (high, moderate, low or very low): for association	Consistent (moderate/ strong evidence): no association	Conflicting/inconsistent, or weak/ limited evidence	High/moderate/low/ very low
**Age**	Six reviews (Harmelink et al. [40], Hernández et al. [42], Lewis et al. [43], Lungu et al. [41], Migliorini et al. [33], Murphy et al. [37])	3/6 (Harmelink et al. [40], Hernández et al. [42], Lewis et al. [43])	0	3/6 (Lungu et al. [41], Migliorini et al. [33], Murphy et al. [37])	2 (Hernández et al. [42], Migliorini et al. [33])	3 (Lewis et al. [43], Lungu et al. [41], Murphy et al. [37])	1 (Harmelink et al. [40])	<u>Low confidence:</u> 2 reviews reporting and association (1 low RoB and 1 high RoB), 3 reported no association (1 low RoB and 2 high RoB) and 1 low RoB reported conflicting/ limited evidence
**BMI**	Seven reviews (Harmelink et al. [40], Haynes et al. [39], Hernández et al. [42], Lewis et al. [43], Lungu et al. [41], Migliorini et	4/7 (Harmelink et al. [40], Hernández et al. [42], Lewis et al. [43],		3/7 (Haynes et al. [39], Lungu et al. [41], Migliorini et al. [33])	2 (Lungu et al. [41], Pozzobon et al. [31])	3 (Haynes et al. [39], Lewis et al. [42], Migliorini et al. [33])	2 (Harmelink et al. [40], Hernández et al. [42])	<u>Low confidence:</u> 3 reviews reporting no association (1 low RoB and 2 high RoB), 2 reported an association (1 low RoB and 1 high RoB) and 2 low RoB reported conflicting/

	al. [33], Pozzobon et al. [31])	Pozzobon et al. [31])					limited evidence or weak association
**Gender**	Six reviews (Harmelink et al. [40], Hernández et al. [42], Lewis et al. [43], Lungu et al. [41], Migliorini et al. [33], Santaguida et al. [45])	3/6 (Harmelink et al. [40], Hernández et al. [42], Lewis et al. [43])	3/6 (Lungu et al. [41], Migliorini et al. [33], Santaguida et al. [45])	2 (Hernández et al. [42], Santaguida et al. [45])	3 (Lewis et al. [43], Lungu et al. [41], Migliorini et al. [33])	1 (Harmelink et al. [40])	Low confidence: 3 review reporting no association (1 low RoB and 2 high RoB), 2 reported an association (1 low RoB and 1 high RoB) and 1 low RoB reported conflicting/ limited evidence or weak association
**Diagnosis**	One review (Hernández et al. [42])	1/1 (Hernández et al. [42])				1 (Hernández et al. [42])	Low confidence: not possible to draw a conclusion (1 low RoB)
**Comorbidiy**	Four reviews (Harmelink et al. [40], Hernández et al. [42], Lewis et al. [43], Lungu et al. [41])	3/4(Harmelink et al. [40], Hernández et al. [42], Lewis et al. [43])	1/4 (Lungu et al. [41])	1 (Lungu et al. [41])	1 (Lewis et al. [43])	2 (Harmelink et al. [40], Hernández et al. [42])	Low confidence: 1 low RoB review reporting no association, 1 high RoB review reported an association and 2 low RoB reported conflicting/limited evidence or weak association

**Diabetes Mellitus**	One review (Harmelink et al. [40])	1/1 (Harmelink et al. [40])				1 (Harmelink et al. [40])	<u>Low confidence:</u> very low evidence for association (1 low RoB)
**Kidney Disease**	Two reviews (Harmelink et al. [40], Podmore et al. [36])	2/2 (Harmelink et al. [40], Podmore et al. [36])			1 (Podmore et al. [36])	1 (Harmelink et al. [40])	<u>Low confidence</u>: 1 low RoB review reporting no association and 1 low RoB reporting very low evidence for association
**Radiographic severity**	One review (Harmelink et al. [40])	1/1 (Harmelink et al. [40])				1 (Harmelink et al. [40])	<u>Low confidence:</u> 1 low RoB review reporting very low evidence for association
**Low back pain**	One review (Hernández et al. [42])	1/1(Hernández et al. [42])				1 (Hernández et al. [42])	<u>Low confidence:</u> not possible to draw a conclusion (1 low RoB)
**Level of education**	Four reviews (Harmelink et al. [40], Hernández et al. [42], Lewis et al. [43], Lungu et al. [41])	3/4 (Harmelink et al. [40], Hernández et al. [42], Lewis et al. [43])	1/4 (Lungu et al. [41])	1 (Lungu et al. [41])	1 (Lewis et al. [43])	2 (Harmelink et al. [40], Hernández et al. [42])	<u>Low confidence:</u> 2 low RoB review reporting no association, 1 high RoB reported association and 2 low RoB reported conflicting/limited evidence or weak association

**Socioeconomic status**	Two reviews (Harmelink et al. [40], Hernández et al. [42])	2/2 (Harmelink et al. [40], Hernández et al. [42])		2 (Harmelink et al. [40], Hernández et al. [42])	<u>Low confidence</u>: 2 low RoB reviews reported conflicting/limited evidence or weak association
**Income**	One review (Harmelink et al. [40])	1/1 (Harmelink et al. [40])		1 (Harmelink et al. [40])	<u>Low confidence:</u> 1 low RoB reported very low evidence for association
**Waiting list**	One review (Hernández et al. [42])	1/1 (Hernández et al. [42])		1 (Hernández et al. [42])	<u>Low confidence:</u> 1 low RoB review reported conflicting/limited evidence or weak association
**Preoperative quadriceps muscle force**	One review (Harmelink et al. [40])	1/1 (Harmelink et al. [40])		1 (Harmelink et al. [40])	<u>Low confidence:</u> 1 low RoB reported very low evidence for no association
**Preoperative flexion contracture**	One review (Harmelink et al. [40])	1/1 (Harmelink et al. [40])		1 (Harmelink et al. [40])	<u>Low confidence:</u> 1 low RoB reported very low evidence for association
**Preoperative ROM**	One review (Harmelink et al. [40])	1/1 (Harmelink et al. [40])		1 (Harmelink et al. [40])	<u>Low confidence:</u> 1 low RoB reported very low evidence for no association

**Preoperative quality of life**	Three reviews (Harmelink et al. [40], Khatib et al. [47], Lungu et al. [41])	1/3 (Harmelink et al. [40])	2/3 (Khatib et al. [47], Lungu et al. [41])	2 (Khatib et al. [47], Lungu et al. [41])		1/3 (Harmelink et al. [40])	Low confidence: 2 high RoB reviews reported association and 1 low RoB reported very low evidence for no association
**Contralateral hip osteoarthritis**	One review (Lungu et al. [41])	1/1 (Lungu et al. [41])		1 (Lungu et al. [41])			Low confidence: 1 high RoB review reported association
**Personality**	Two reviews (Hernández et al. [42], Vissers et al. [46])	1/1 (Hernández et al. [42])	1/1 (Vissers et al. [46])			2 (Hernández et al. [42], Vissers et al. [46])	Low confidence: 2 reviews reported conflicting/limited evidence or weak association (1 low RoB and 1 high RoB)
**Purpose in life**	Two reviews (Khatib et al. [47], Vissers et al. [46])		2/2 (Khatib et al. [47], Vissers et al. [46])	1 (Khatib et al. [47])	1 (Vissers et al. [46])		Low confidence: 1 high RoB review reported no association and 1 high RoB reported an association
**Emotionally**	One review (Vissers et al. [46])		1/1 (Vissers et al. [46])		1 (Vissers et al. [46])		Low confidence: 1 high RoB review reporting no association

**Self-Efficacy**	Two reviews (Hernández et al. [42], Vissers et al. [46])	1/2 (Hernández et al. [42])	1/2 (Vissers et al. [46])	1 (Vissers et al. [46])		1 (Hernández et al. [42])	<u>Low confidence:</u> 1 high RoB review reported association and 1 low Rob review reporting conflicting/limited
**Psychological distress**	One review (Harmelink et al. [40])	1/1 (Harmelink et al. [40])				1 (Harmelink et al. [40])	<u>Low confidence:</u> 1 low RoB review reported conflicting/limited evidence or weak association
**Patient expectations**	One review (Hernández et al. [42])	1/1 (Hernández et al. [42])				1 (Hernández et al. [42])	<u>Low confidence:</u> 1 low RoB review reported conflicting/limited evidence or weak association

BMI: Body Mass Index / RoB: Risk of bias
